# Honokiol ameliorates reserpine-induced fibromyalgia through antioxidant, anti-inflammatory, neurotrophic, and anti-apoptotic mechanisms

**DOI:** 10.1038/s41598-025-07209-w

**Published:** 2025-07-17

**Authors:** Suzan A. Khodir, Eman M. Sweed, Hala El-Haroun, Tarek H. Abd-Elhamid, Sara A. El Derbaly, Amany R. Mahmoud, Shaimaa M. Motawea

**Affiliations:** 1 Medical Physiology Department, Faculty of Medicine, Menoufia University, Menoufia, 32511 Egypt; 2 Medical Physiology Department, Menoufia National University, Menoufia, Egypt; 3 Clinical Pharmacology Department, Faculty of Medicine, Menoufia University, Menoufia, 32511 Egypt; 4 Clinical Pharmacology Department, Menoufia National University, Menoufia, Egypt; 5 Histology and Cell Biology, Faculty of Medicine, Menoufia University, Menoufia, 32511 Egypt; 6 AlRyada University for Science and Technology, Menoufia, 32511 Egypt; 7 Histology and Cell Biology, Faculty of Medicine, Assiut University, Assiut, 71515 Egypt; 8 Anatomy and Histology, Faculty of Medicine, Aqaba Medical Sciences University, Aqaba, 77110 Jordan; 9 Medical Biochemistry and Molecular Biology Department, Faculty of Medicine, Menoufia University, Menoufia, 32511 Egypt; 10 Human Anatomy and Embryology, Faculty of Medicine, Assiut University, Assiut, 71515 Egypt; 11 Anatomy and Histology, College of Medicine, Qassim University, Buraydah, Saudi Arabia

**Keywords:** Caspase-3, CGRP, Fibromyalgia, Honokiol, JAK/STAT3, Prostaglandin E2, Biochemistry, Neurology

## Abstract

Fibromyalgia (FM) is a chronic condition characterized by widespread musculoskeletal pain, fatigue, psychological disturbances, and sleep issues. Honokiol (HNK) is a bioactive compound known for its medicinal properties. This study evaluated HNK’s effectiveness in alleviating pain, depression, and anxiety in a reserpine-induced FM rat model. Thirty male rats were divided into three groups: control, RES (FM-induced), and RES + HNK. HNK was supplemented to RES + HNK in a dose of 8 mg/kg for 21 days. Behavioral assessments included the open field, elevated plus maze, and forced swim tests, while pain was evaluated using treadmill endurance, tail flick latency, paintbrush, and rotarod tests. Brain homogenates were analyzed for neurotransmitters, antioxidants, pro-inflammatory cytokines, and gene expressions. Histopathological evaluation of spinal cords assessed markers of inflammation and apoptosis. Results showed that HNK administration improved behavior and reduced pain. This was linked to reduced levels of malondialdehyde, tumor necrosis factor-α, and prostaglandin E2, alongside increased superoxide dismutase and interleukin-10. Additionally, HNK downregulated the expression of calcitonin gene-related peptide and the JAK/STAT3 gene. These findings suggest that HNK alleviates FM symptoms through its antioxidant, anti-inflammatory, neuroprotective, and anti-apoptotic properties, indicating its potential as a therapeutic agent for FM.

## Introduction

Fibromyalgia (FM) is a medical disorder characterized by persistent musculoskeletal pain. Key symptoms include muscle and joint stiffness, insomnia, fatigue, depression, anxiety, mood disturbances, cognitive impairment, generalized sensitivity, and difficulty performing daily tasks^1^. Fibromyalgia (FM) affects around 2.7% of the global population, leading to significant morbidity and disability, particularly among women^2^.

The condition is complex, involving multiple contributing factors. Persistent oxidative stress and central sensitization are two primary mechanisms implicated in its development and persistence^3^. Despite significant advancements in understanding FM’s pathophysiology, its exact cause remains unknown^4^.

FM is primarily associated with dysfunctions in the transmission of monoaminergic neurotransmitters. This leads to elevated levels of excitatory neurotransmitters, such as glutamate and substance P, alongside reduced levels of serotonin and norepinephrine in the descending anti-nociceptive pathways of the spinal cord. Additional abnormalities include dopamine dysregulation and impaired function of endogenous brain opioids. Together, these factors seem to underpin the core pathophysiology of FM^1^. Furthermore, an imbalance between anti-inflammatory and pro-inflammatory cytokines may be a potential peripheral pathological factor in the etiopathogenesis of FM^5^.

Neurogenic inflammation occurs when sensory neurons trigger inflammation by releasing inflammatory neuropeptides. The stimulation of calcitonin gene-related peptide (CGRP) leads to the release of additional neuropeptides and neurotrophins, which further activate nerve fibers by attracting inflammatory cells, thereby intensifying the inflammatory response^6^.

Central sensitization, which is thought to play a role in FM pathogenesis, is responsible for the increase in excitatory neurotransmitters such as calcitonin gene-related peptide (CGRP), glutamate and substance P, or a decrease in inhibitory neurotransmitters such as serotonin and norepinephrine^7^.

The Janus kinase/signal transducers and activators of transcription (JAK/STAT) pathway could modulate anti-nociceptive mechanisms since IL-10, a potent anti-inflammatory cytokine, carries out its action through the JAK/STAT pathway^8^. STAT3 plays a vital role in nociceptive transmission^9^.

Honokiol (HNK) is a naturally occurring pleiotropic lignan extracted from the *Magnolia grandiflora* species, widely known in Japan and used in traditional medical practices across much of Asia^10^.

HNK is a polyphenolic compound known for its diverse therapeutic effects, including anxiolytic, antidepressant, antithrombotic, antimicrobial, antispasmodic, analgesic, neuroprotective, and anti-tumorigenic properties^11^. Its high lipophilicity allows it to cross the blood-brain and blood-cerebrospinal fluid barriers, making it effective in treating central nervous system (CNS) disorders. Studies suggest honokiol protects against oxidative damage, neuroinflammation, and excitotoxicity while supporting mitochondrial function^12,13^. It has shown promise in improving neuronal and motor impairments in Parkinson’s disease, enhancing cognitive function in APP/PS1 models, and reducing cerebral edema and neurobehavioral deficits after subarachnoid hemorrhage^13,14^.

This study aimed to investigate the potential role of honokiol in alleviating pain, depression, and anxiety in a reserpine-induced fibromyalgia model in adult male albino rats, as well as to explore the underlying mechanisms through which it exerts these effects.

## Materials and methods

### Animals

Thirty adult male Wistar albino rats (Tanta Center for Laboratory Animals, Tanta, Egypt), each weighing between 180 and 250 g, were used in this study. The rats were housed under controlled conditions with a natural 12:12 light-dark cycle, given unlimited access to water, and fed a standard rat diet. They were allowed to acclimate to their environment for one week prior to the start of the investigation. The study was approved by the Research Ethics Committee of the Faculty of Medicine at Menoufia University, Egypt (IRB Number: 5-2024Bio-6). All experiments adhered to the Animals in Research: Reporting In Vivo Experiments (ARRIVE) standards^15^. All experiments were performed in accordance with relevant guidelines and regulations.

The sample size was determined using the Resource Equation Approach, calculating the maximum number of rats needed (*n* = 20/K + 1), where K is the number of groups and n is the number of subjects per group. The calculated sample size per group was 7 rats, which was increased to 10 rats to account for a potential 25% dropout rate. The study was designed with an 80% power and a 95% confidence level.

### Experimental design

The rats were evenly divided into three groups, with each group consisting of ten rats:

Control Group: Rats in this group received a daily subcutaneous (s.c.) injection of 0.5% acetic acid at a dose of 1 ml/kg of body weight for three consecutive days and concomitantly intragastric administration of a 5% dimethyl sulfoxide (DMSO) solution at a volume of 10 ml/kg for twenty-one consecutive days.

Reserpine-Induced Fibromyalgia Group (RES Group): A fibromyalgia (FM) model was induced in this group by administering a daily s.c. injection of reserpine (Sigma-Aldrich Chemical Co., St. Louis, MO, USA) at a dose of 1 mg/kg, dissolved in 0.5% acetic acid, for three consecutive days, following the method described by Nagakura et al.^16–18^. Concomitantly starting from the 1st day of the study period rats received intragastric administration of a 5% DMSO solution at a volume of 10 ml/kg for twenty-one consecutive days.

The Open Field Test (OFT), Elevated Plus Maze (EPM) Test, and Forced Swim Test (FST) were used to confirm the successful induction of reserpine-induced FM.

Reserpine-Induced Fibromyalgia Treated with Honokiol (RES + HNK Group): Rats in this group were treated as in the RES group as in previous group. Concomitantly honokiol (HNK) supplementation (Sigma-Aldrich, St. Louis, MO, USA), dissolved in a 5% DMSO solution at a concentration of 8 mg/kg at a volume of 10 ml/kg were given to rats staring from the 1st day of study period continuous for twenty-one days. The HNK was administered intragastrically for twenty-one days^19^.

The reserpine-induced fibromyalgia model was chosen because reserpine causes FM-related pathophysiological and behavioral alterations (nociceptive, anxiety-like, and depressive-like) and has the potential for use in widespread chronic pain investigations^20^. Furthermore, reserpine causes FM-like symptoms in both female and male animals^20,21^. The reserpine-induced FM model is well-established, effectively mimicking FM’s clinical and etiological features, including reduced biogenic amines, depression-like symptoms, and musculoskeletal pain^22^.

Rats were subjected to OFT, EPM and FST on day 19th of the study with 2 h interval. Tail Flick Latency Test followed by Paintbrush Test were performed on the 20th day of the study with 2 h interval between each test. On day 21st of the study period rats were subjected to rotarod performance test followed by Treadmill Exercise Endurance Test with 2 h interval between each test.

After completing the behavioral tasks, the rats were euthanized using ketamine (100 mg/kg) and xylazine (10 mg/kg, intraperitoneal) anesthesia. Their brains were then harvested, washed with saline, dried, and weighed. Each brain was divided into two halves: the first half was used for histological and immunohistochemical analyses, and the second half was frozen in liquid nitrogen and stored at -80 °C for biochemical examination.

**Fig. 1:**
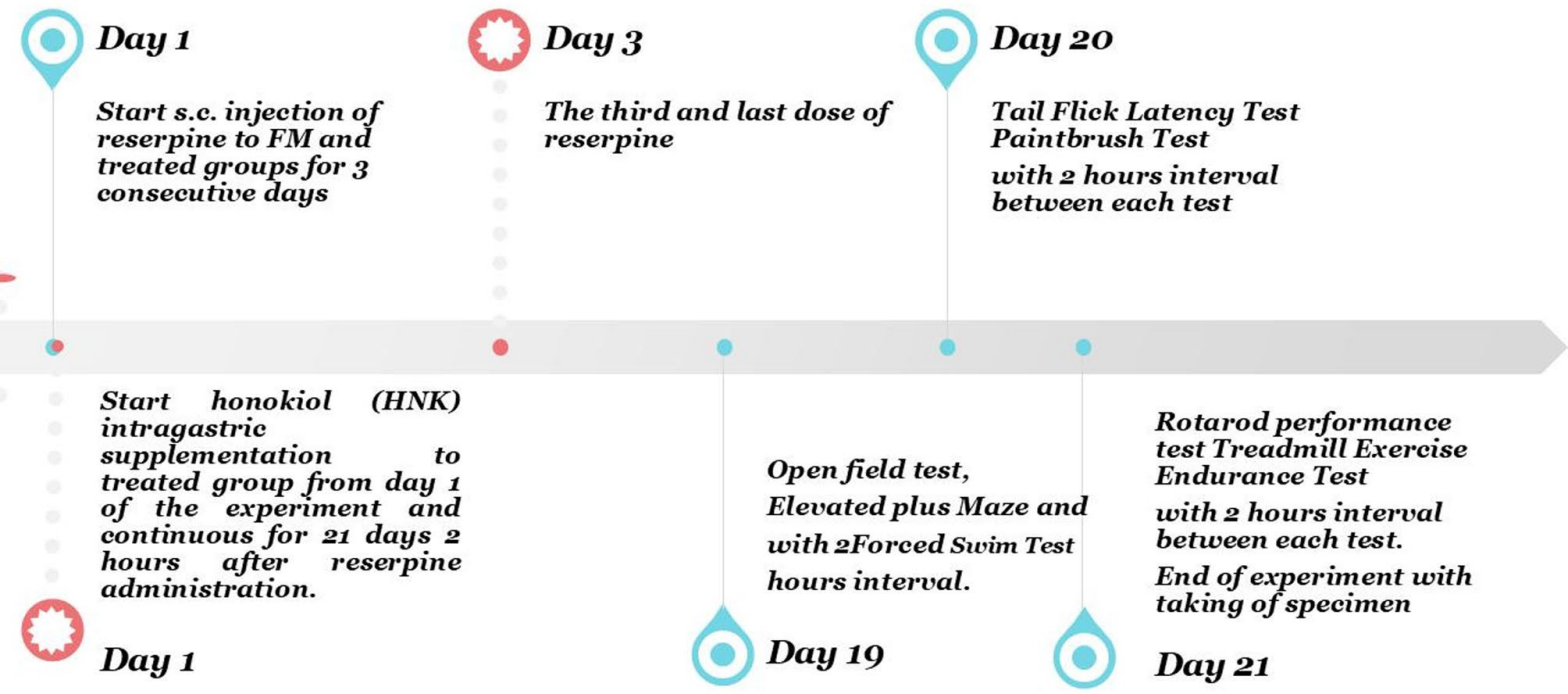
Schematic presentation of study design.

The rats’ body weight (BW) was recorded at the end of the experiment. BW was measured using a digital balance, ensuring accuracy by calibrating the scale before each measurement. The unit of measurement was grams (Fig. 1).

### Behavioral assessment

#### Open Field Test (OFT)

OFT was used to assess the rat’s locomotor’s activity. A wooden arena measuring 100 × 100 × 60 cm, divided into twenty-five squares (each 20 cm), was used for the Open Field Test. Each rat was placed in the center of the arena and allowed to explore freely for 15 min. The latency to leave the starting position in seconds and the frequency of ambulation (number of squares crossed) were recorded for each rat^23^.

#### Elevated Plus Maze (EPM) Test

EPM was used to assess the anxiety like behavior in rats. The Elevated Plus Maze, a plus-shaped apparatus, was used to assess anxiety-like behavior in rats^23^. Each rat was placed in the central area of the maze and given 10 min to explore. An overhead camera recorded the rats’ movements, and the time spent in the open arms of the maze was measured. Anxiety-like behavior was inversely related to the amount of time spent in the open arms in seconds were calculated by using stopwatch.

#### Forced Swim Test (FST)

FST was used to assess the depressive behavior in rats. All groups underwent the Forced Swim Test, with slight modifications. Each rat was individually placed in a glass tank (30 cm in diameter × 40 cm deep) filled with 25 cm of water at 25 °C. During the test, the duration of immobility—defined as the minimal movement required to keep the rat’s head above water—was measured in seconds over a period of 5 min, along with the latency to the first instance of immobility^24^.

### Pain assessment

#### Tail Flick latency test

Tail Flick Latency Test is one of the tests used to assess pain threshold in rats. The Tail Flick Latency Test was conducted using a tail flick apparatus (Harvard Apparatus, USA) to measure thermal nociceptive response. The rats were gently handled, and their tails were exposed to radiant heat at a point 3 cm from the tip. The latency for the rat to flick its tail (tail-flick latency) was recorded in seconds. To prevent tissue damage, the heat stimulus was terminated after ten seconds (cutoff latency)^25^.

#### Paintbrush test

Paintbrush Test is one of the tests used to assess pain. Rats were placed in plastic cages with wire mesh floors. A soft paintbrush was used to gently stroke the plantar surface of the hind paw five times at five-second intervals, from heel to toe. The number of withdrawal responses (0–5) was recorded. This stimulus typically does not elicit a response in normal rats, so any withdrawal is considered indicative of mechanical dynamic allodynia. The test was repeated three times, with a five-minute interval between each trial. Each rat’s total score was recorded, ranging from 0 to 15 relaying on the sum of the number of withdrawal responses in the 3 trials to get a single cumulative score for dynamic allodynia^26^.

#### Rotarod performance test

Rotarod Performance Test is one of the tests used to assess pain and coordination in rats. The Rotarod Performance Test was used to measure motor coordination and balance. Rats were subjected to an accelerating rotarod, with the speed increasing from 5 to 16 rpm over one minute and then maintained at that speed. The time until the first fall was recorded, with a maximum cutoff time of three minutes. The data is expressed as the time to fall in seconds^27^.

#### Treadmill exercise endurance test

The Treadmill Exercise Endurance Test is one of the tests used to assess pain. Rats were trained to run at a speed of 10 m per minute for four days. Twenty-four hours later, they were required to run at 15 m per minute on a motorized treadmill until exhaustion. Exhaustion was defined as the inability to continue running after being placed at the front of the treadmill three times. The average time to fatigue for each rat was recorded in minutes^28^.

### Biochemical assessment

Brain tissue homogenates were prepared by individually homogenizing weighed brain specimens using a tissue homogenizer (MPW Medical Instruments, MPW120, China). Calorimetric kits from the Biodiagnostic Company (Dokki, Giza, Egypt) were used to measure brain levels of malonaldehyde (MDA) and superoxide dismutase (SOD). Levels of dopamine, serotonin, norepinephrine, tumor necrosis factor (TNF)-α, prostaglandin (PG)-E2, and interleukin (IL)-10 in the brain were determined using corresponding rat ELISA kits: Dopamine (201-11-0220, Sunred CO., Shanghai, China), Serotonin (201-12-1712, Sunred, Shanghai, China), Norepinephrine (201-11-3359, Sunred, Shanghai, China), TNF-α (Saint Charles, Missouri, USA, Assaypro LLC.), PG-E2 (ab133021, Abcam, Cambridge, UK), and IL-10 (ab214566, Abcam, Cambridge, UK), following the manufacturer’s instructions.

### Real-time (RT)-PCR analysis

Brain specimens were subjected to RT-PCR analysis to quantify the relative expression of CGRP, JAK, and STAT3. Total RNA was extracted using the miRNeasy Mini Kit (Qiagen, USA) and assessed for quality and purity with a Nanophotometer N60 (Implen, Germany). Reverse transcription was performed using a QuantiTect reverse transcription kit (Qiagen, USA) on a programmable thermocycler (Biometra, Germany). Amplification of cDNAs was achieved using the QuantiTect SYBR Green PCR Kit (Qiagen, USA), with GAPDH primers serving as an internal control. A standard cycling protocol was followed, consisting of initial denaturation at 95 °C for 15 min, followed by 45 cycles of denaturation (94 °C, 15 s), annealing (55 °C, 30 s), and extension (72 °C, 30 s). Melting curve analysis was conducted to ensure amplification specificity and eliminate primer dimer formation. The 7500 real-time PCR system (Applied Biosystems, USA) was used to analyze the data, and the ΔΔCt method was employed to calculate relative gene expression levels^29^. The primers used were as follows:

**Table:** 

CGRP	F:5’ TCTAGTGTCACTGCCCAGAAGAGA-3’ *R*:5’-GGCACAAAGTTG TCCTTCACCACA-3’
JAK	F: 5’GAGGAATGTACTGGGCGTCT-3’ R:5’-TGCAGCCGGAGAGACATTTT-3’
STAT3	F: 5’ATGTCTCAAGATGGCGGAGC-3’ R:5’-GACCGACAGCCAGTCAAAGA-3’
GAPDH	F: 5’-CCACTCCTCCACCTTTGAC-3’ R: 5’-ACCCTGTTGCTGTAGCCA − 3’

### Histopathological examinations

#### Histological study

Cerebral cortex sections were stained with hematoxylin and eosin (H&E) for general histological evaluation. Paraffin sections were deparaffinized and rehydrated prior to immunohistochemical staining.

#### Immunohistochemical Study

**Glial Fibrillary Acidic Protein (GFAP) (Gliosis)**: Astrocytes were identified using a monoclonal mouse antibody against GFAP (Cat. no. ab7260; Abcam, Inc., Cambridge, MA, USA 1:400 in PBS). The peroxidase-labeled streptavidin-biotin method and diaminobenzidine (Dakopatts, Glostrup, Denmark) were employed for visualization. Slides were counterstained with Mayer’s hematoxylin^30^.

**CD68**: Microglia were detected by anti-CD68 mouse monoclonal antibody (AMAB90873; Sigma-Aldrich Chemie GmbH; 1:1000 wits PBS) using peroxidase-labeled streptavidin-biotin. Slides were cleaned using distilled water. Later, sections were counter-stained with Mayer’s hematoxylin^30^.

**Caspase-3 (Apoptosis Marker)**: A 1:50 dilution of anti-caspase-3 (rabbit polyclonal antibody, Fermont, CA 94539, Thermo Science, USA) was utilized. The 1ry antibody utilized for caspase-3 was a ready-to-use rabbit polyclonal antibody (CAT-No. RB-3425-R2) [18].

Normal lymphoid tissue served as a positive control for caspase-3, while liver Kupffer cells were used as a positive control for CD68. Negative controls omitted the primary antibody.

### Statistical analysis

Data analysis was conducted using SPSS version 27 (SPSS, Inc., USA). The Shapiro-Wilk test was employed to assess normality of data distribution. For normally distributed data, mean ± standard deviation was reported. One-way ANOVA followed by Tukey’s post-hoc test was used to compare group differences. For non-normally distributed data, median ± interquartile range (IQR) were presented, and the Kruskal-Wallis test was utilized to assess group differences. Cohen’s d statistic was calculated to compare the effect sizes. Eta-squared represented the effect size. A p-value less than 0.05 was considered statistically significant.

## Results

### Body weight change

**Fig. 2:**
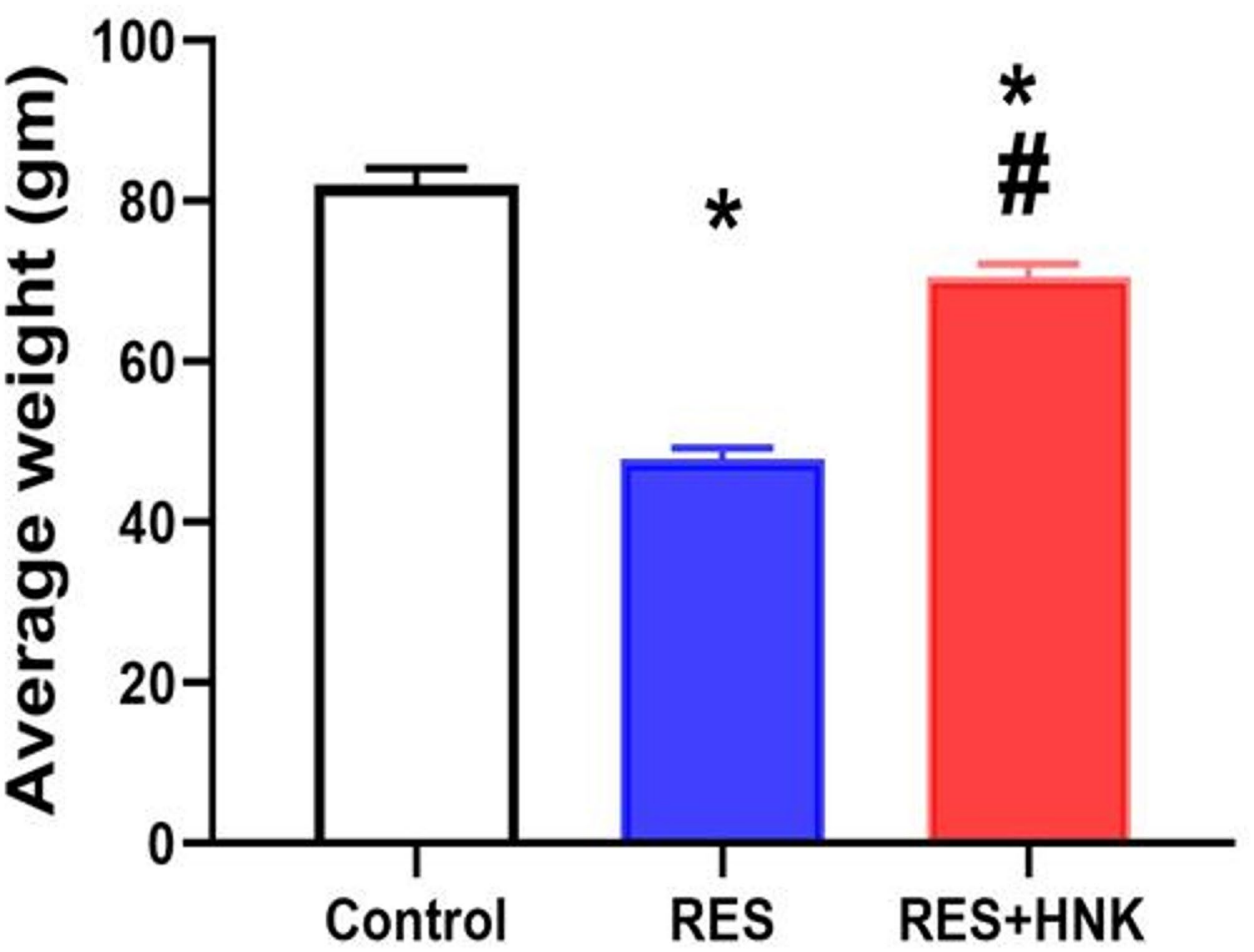
Honokiol impact on body weight in the RES group. Data are defined as mean ± standard deviation (number equals ten). Group comparisons were made using the ANOVA test. * P-value is less than 0.05 vs. the control group. # P-value is less than 0.05 vs. the RES group RES: reserpine, HNK: honokiol. No of rats in each group was 10.

The body weight of the RES group was significantly lower than that of the control group (47.9 ± 1.4 vs. 82.08 ± 2.01 gm, respectively; *P* < 0.05). In contrast, the body weight of the RES + HNK group was significantly higher than that of the RES group (70.5 ± 1.7 g; P-value less than 0.05). But the RES + HNK group’s body weight was still much lower than the control group’s (P-value less than 0.05). The effect size was large regarding body weight (0.988) (Fig. 2).

### Behavioral assessment

#### OFT

**Fig. 3:**
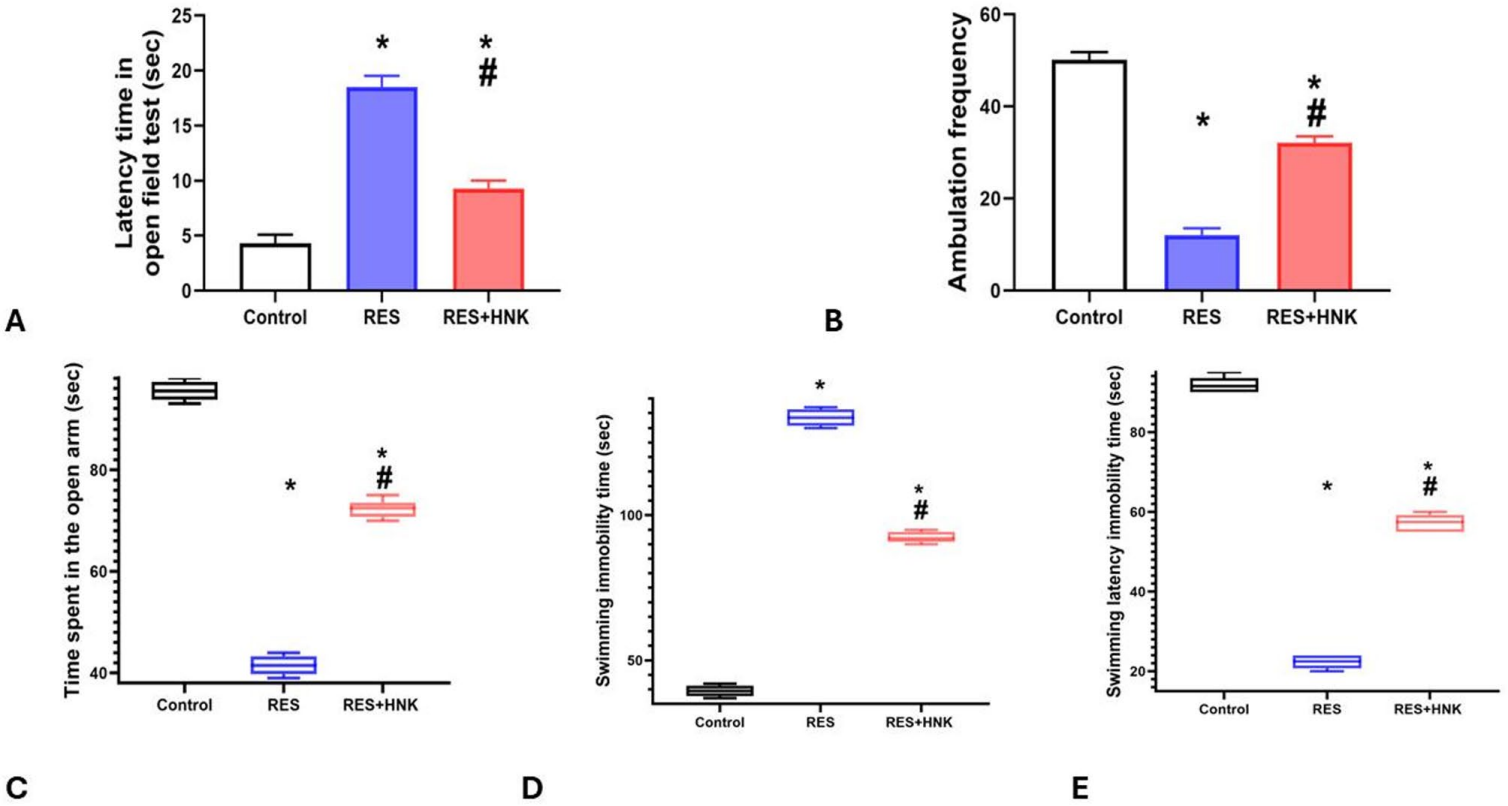
Honokiol impact on the latency time in the open field test (OFT) (**A**), Ambulation frequency (OFT) (**B**), duration in the open arm in the elevated plus maze (EPM) test (**C**), immobility time in the forced swimming test (FST) (**D**), and immobility latency in FST in the RES group. Data are defined as mean ± standard deviation for open field test. Group comparisons were made using the ANOVA test in OFT. While the Kruskal Wallis test was used to compare groups in EPM, and FST and data represented as median ± IQR. * P-value is less than 0.05 vs. the control group. # P-value is less than 0.05 vs. the RES group *RES* reserpine, *HNK* honokiol. No of rats in each group was 10.

According to behavioral assessment tests, the RES group’s latency time was significantly higher than that of the control group (18.5 ± 1.04 vs. 4.3 ± 0.8 s, respectively; P-value less than 0.05). On the other hand, the latency of the RES + HNK group was significantly lower than that of the RES group (9.3 ± 0.7 s; P-value less than 0.05). Nonetheless, it continued to rise significantly in comparison to the controls (P-value < 0.05). Furthermore, ambulation frequency (squares crossed) was significantly lower in the RES group than in the control group (12.1 ± 1.4 vs. 50.1 ± 1.7, respectively; P-value less than 0.05). On the other hand, the frequency of ambulation was significantly higher in the RES + HNK group than in the RES group (32.1 ± 1.4; P-value less than 0.05). Nevertheless, it was still significantly less than that of the controls (P-value less than 0.05) The effect size was large regarding open field latency and ambulation (0.981, 0.992 respectively) (Fig. 3A,B).

#### EPM

When compared to the controls, the RES group spent significantly the median ± IQR time on the EPM’s open arm was (41.5 ± 3.5 vs. 95.5 ± 3.5 s, respectively; P-value less than 0.05). However, it was considerably greater in the RES + HNK group (72.5 ± 3.75 s; P-value less than 0.05) than in the RES group. It was still much lower than the controls, though (P-value < 0.05). The effect size was large regarding time spent in the open arm (0.994) (Fig. 3C).

#### FST

When compared to the controls, the RES group’s the median ± IQR immobility time increased significantly (133.5 ± 5.5 vs. 39.5 ± 3.5 s, respectively; P-value less than 0.05). Conversely, the RES + HNK group experienced a substantial decrease in comparison to the RES group (92.00 ± 3.5 s; P-value less than 0.05). However, it continued to be considerably higher than the controls (P-value < 0.05). Additionally, the median ± IQR latency to immobility was lower in the RES group than in the controls (22.5 ± 3.5 vs. 91.5 ± 3.5 s, respectively; P-value less than 0.05). But compared to the RES group, it was considerably greater in the RES + HNK group (57.5 ± 4.25 s; P-value less than 0.05). It was still much lower than the controls, though (P-value < 0.05). The effect size was large regarding immobility time and latency to immobility (0.997 and 0.996 respectively) (Fig. 3D, E).

### In terms of pain assessments

#### Treadmill exercise endurance test

**Fig. 4:**
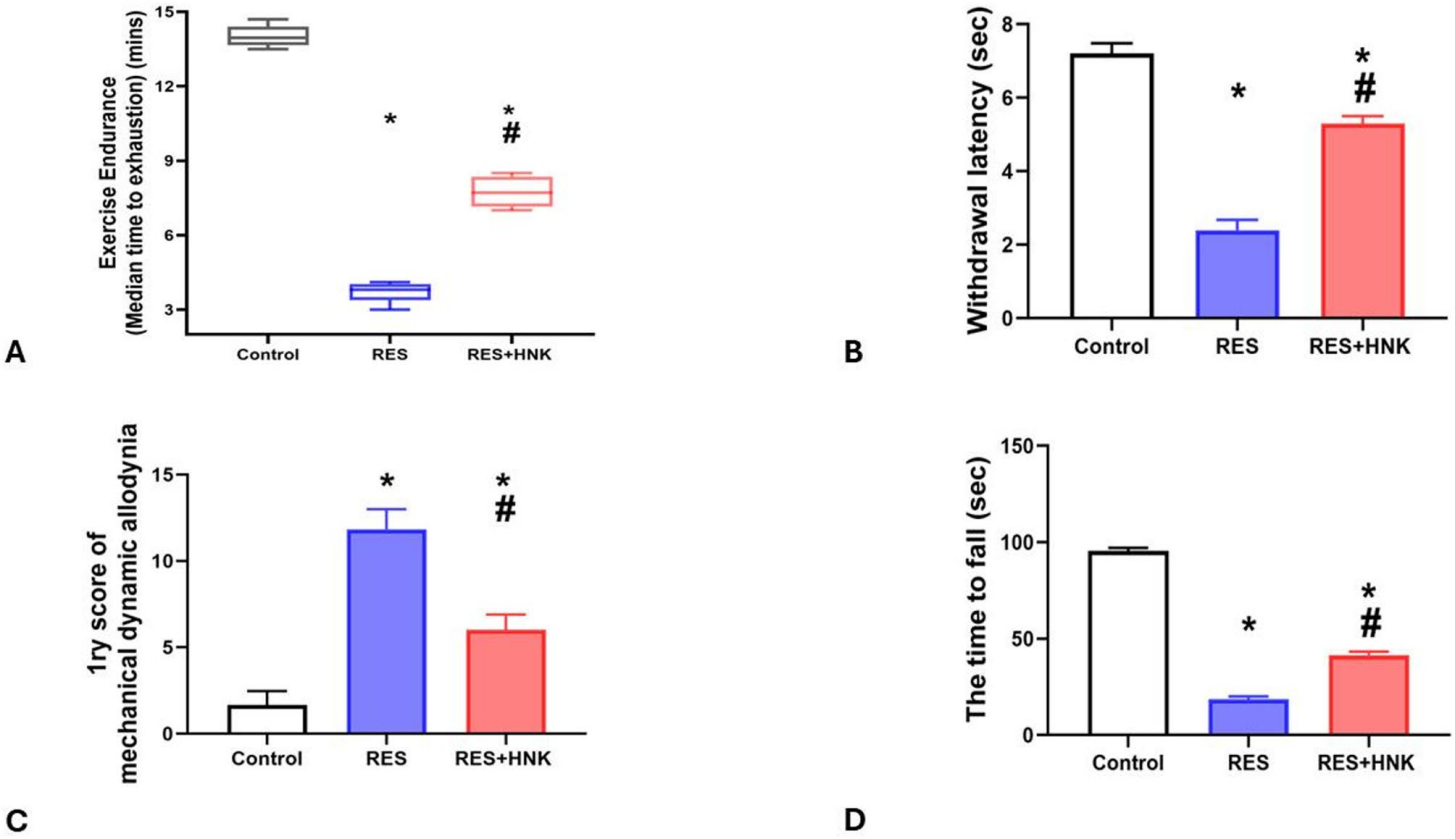
Honokiol impact on the average time till fatigue in the treadmill exercise endurance test described as median ± IQR and group comparison done by Kruskal Wallis test (**A**), while the withdrawal latency in the tail flick latency test (**B**), the main score of mechanical dynamic allodynia (**C**), and the time to fall in rotarod test in the RES group. Data are described as mean ± standard deviation. Group comparisons were made using the ANOVA test. *P-value is less than 0.05 vs. the control group. ^#^P-value is less than 0.05 vs. the RES group. *RES* reserpine, *HNK* honokiol. No of rats in each group was 10.

The median ± IQR time to exhaustion for the RES group was considerably less than that of the controls (3.80 ± 0.65 vs. 13.95 ± 0.85 min, respectively; P-value less than 0.05). But compared to the RES group, it was considerably higher in the RES + HNK group (7.70 ± 1.20 min; P-value less than 0.05). It was still much lower than in the controls, though (P-value < 0.05). The effect size was large regarding exercise endurance time (0.989) (Fig. 4A).

#### The tail flick

Compared to the controls, the RES group’s withdrawal latency was considerably lower (2.40 ± 0.28 vs. 7.21 ± 0.27 s, respectively; P-value less than 0.05). In contrast to the RES group, the RES + HNK group showed a statistically significant increase (5.30 ± 0.20 s; P-value less than 0.05). However, it remained considerably lower than the controls (P-value < 0.05). The effect size was large regarding withdrawal latency (0.986) (Fig. 4B).

#### Mechanical dynamic allodynia

For mechanical dynamic allodynia, the RES group’s primary score was considerably greater than the controls’ (11.83 ± 1.16 vs. 1.66 ± 0.81, respectively; P-value less than 0.05). However, compared to the RES group, it was considerably lower in the RES + HNK group (6.0 ± 0.89; P-value less than 0.05). It was still considerably higher than the controls (P-value < 0.05). The effect size was large regarding mechanical allodynia (0.957) (Fig. 4C).

Rotarod test of performance in comparison to the controls, the RES group spent considerably less time on the rotarod (18.50 ± 3.25 vs. 95.50 ± 2.25 s, respectively; P-value less than 0.05). But compared to the RES group, it was noticeably higher in the RES + HNK group (41.50 ± 3.25 s; *P* < 0.05). However, it remained significantly less than in the controls (P-value less than 0.05) The effect size was large regarding rotarod performance test (0.998) (Fig. 4D).

### Biochemical assessment

#### Neurotransmitter testing

**Fig. 5:**
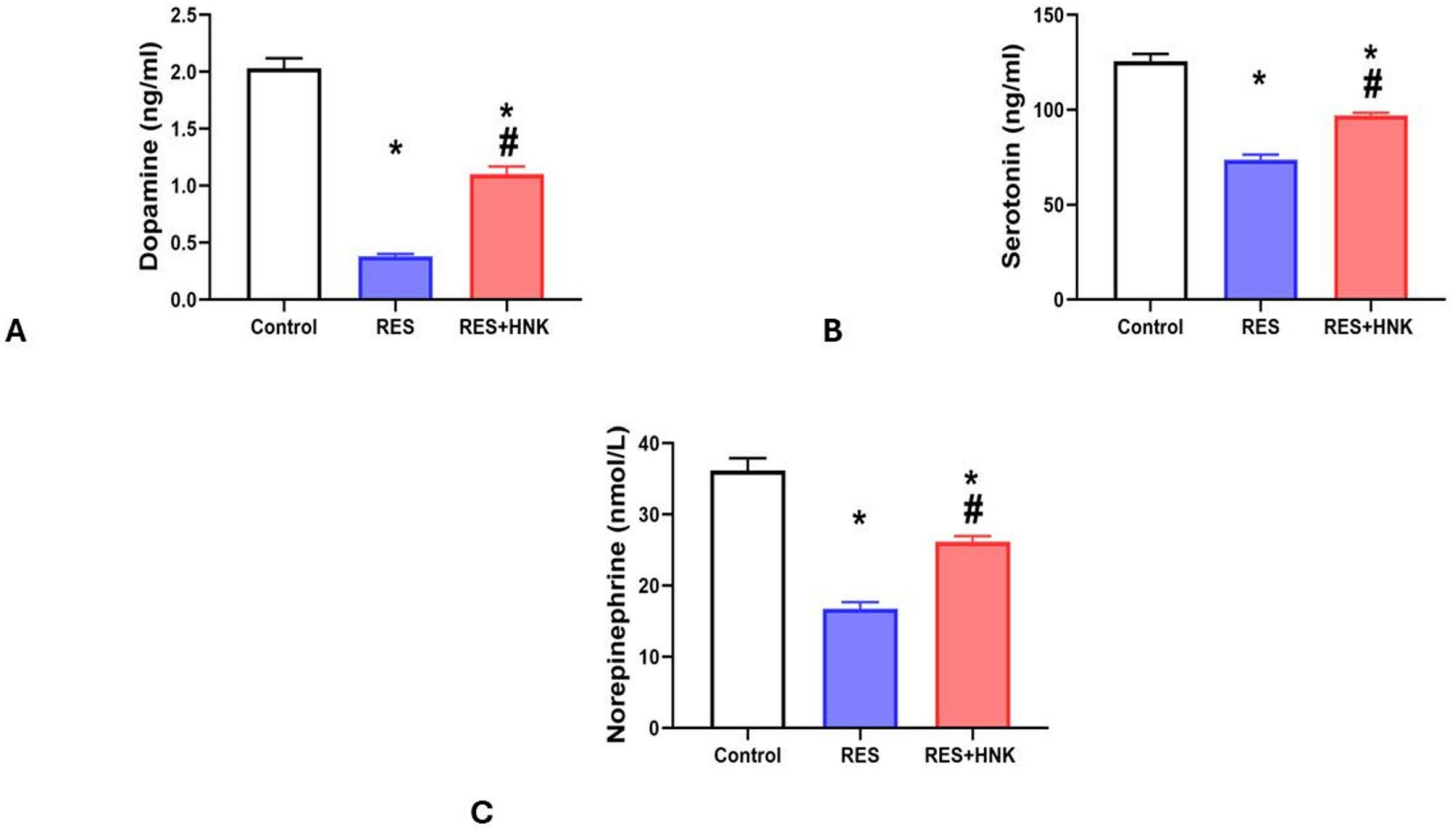
Honokiol impact on brain dopamine level (**A**), brain serotonin level (**B**), and brain norepinephrine level (**C**) in the RES group. Data are described as mean ± standard deviation (number equals ten). Group comparisons were made using the ANOVA test. *P-value is less than 0.05 vs. the control group. # P-value is less than 0.05 vs. the RES group. *RES* reserpine, *HNK* honokiol. No of rats in each group was 10.

Brain dopamine, serotonin, and norepinephrine levels were substantially lower in the RES group than in the controls (0.38 ± 0.02 ng/ml, 73.83 ± 2.63 ng/ml, and 16.75 ± 0.93 nmol/L vs. 2.03 ± 0.09 ng/ml, 125.67 ± 3.77 ng/ml, and 36.16 ± 1.75 nmol/L, respectively; P-value less than 0.05). It was significantly higher in the RES + HNK group (1.10 ± 0.07 ng/ml, 96.91 ± 1.53 ng/ml, and 26.15 ± 0.82 nmol/L; P-value less than 0.05) than in the RES group. It was still significantly lower than in the controls, but (P-value less than 0.05). The effect size was large regarding brain dopamine, serotonin and norepinephrine (0.991, 0.986, and 0.980 respectively) (Fig. 5A–C).

#### Oxidative stress measurements

**Fig. 6:**
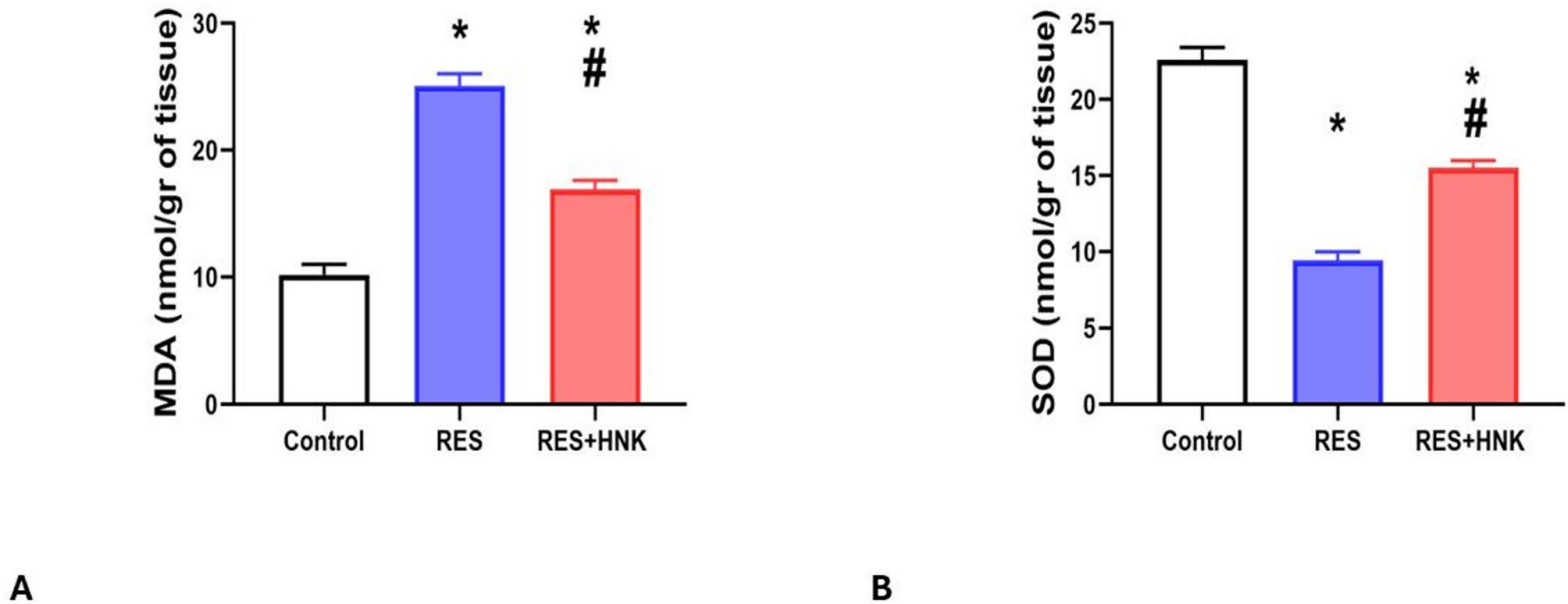
Honokiol impact on brain MDA level (**A**), and brain SOD level (**B**) in the RES group. Data are described as mean ± standard deviation (number equals ten). Group comparisons were made using the ANOVA test. * P-value is less than 0.05 vs. the control group. ^#^P-value is less than 0.05 vs. the RES group. RES: reserpine, HNK: honokiol. No of rats in each group was 10.

In comparison to the controls, the RES group’s brain MDA levels were considerably higher (25.08 ± 0.97 vs. 10.20 ± 0.82 nmol/gr of tissue, respectively; P-value less than 0.05). Compared to the RES group, the RES + HNK group’s levels were significantly lower (16.95 ± 0.68 nmol/gr of tissue; P-value less than 0.05). It was still significantly greater than the controls, nevertheless (P-value < 0.05). The effect size was large (0.984) (Fig. 6A).

Significantly reduced brain SOD levels were also seen in the RES group compared to the control group (9.43 ± 0.55 vs. 22.61 ± 0.82 nmol/g of tissue, respectively; P-value less than 0.05). Compared to the RES group, the RES + HNK group exhibited a statistically significant increase (15.50 ± 0.48 nmol/gr of tissue; P-value less than 0.05). It was still much lower than the controls, though (P-value < 0.05). The effect size was large (0.988) (Fig. 6B).

#### Inflammatory markers

**Fig. 7:**
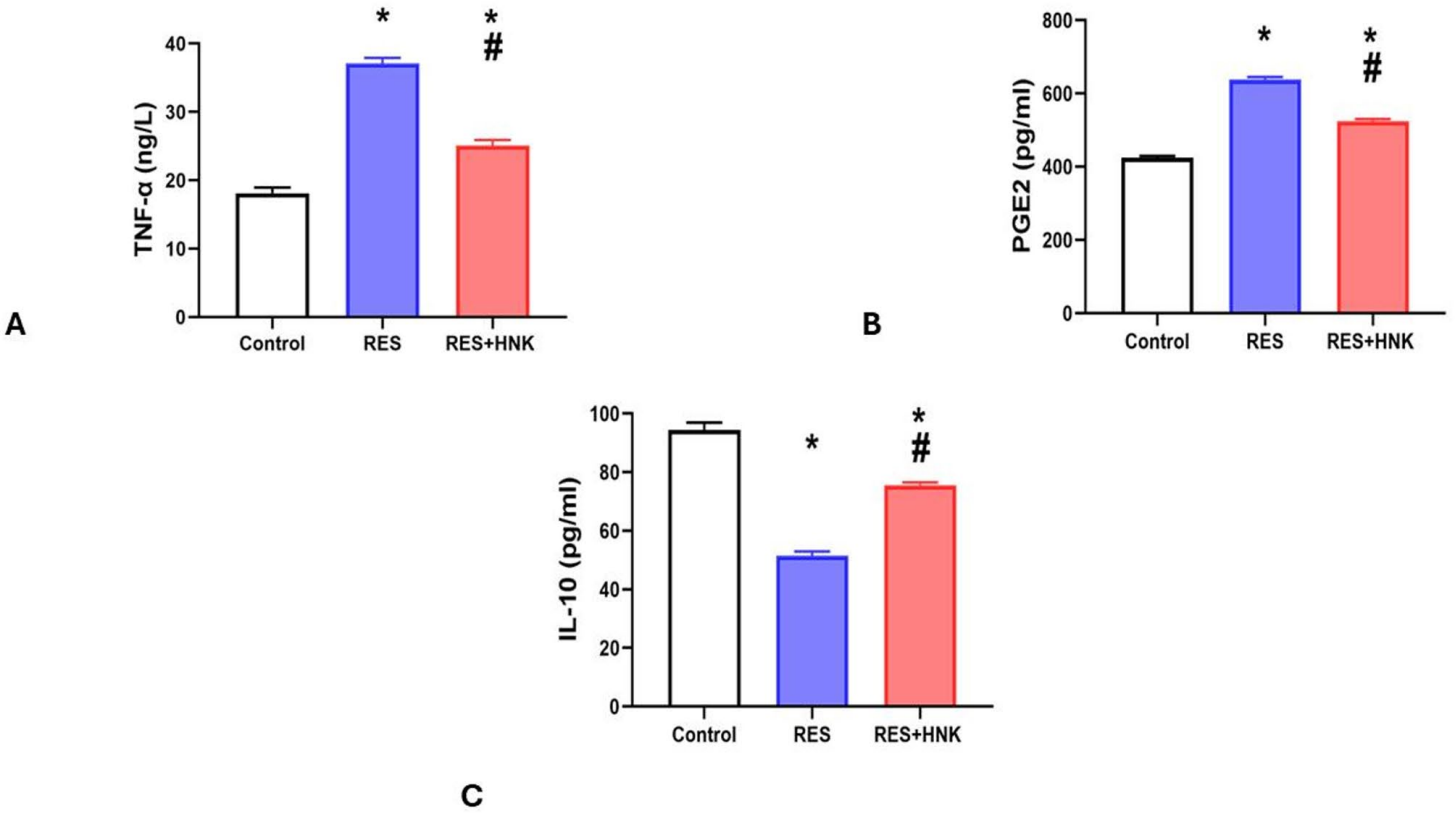
Honokiol impact on brain TNF-α level (**A**), brain IL-10 levels (**B**), and brain PGE2 level (**C**) in the RES group. Data are described as mean ± standard deviation (number equals ten). Group comparisons were made using the ANOVA test. *P-value is less than 0.05 vs. the control group. # P-value is less than 0.05 vs. the RES group. *RES* reserpine, *HNK* honokiol. No of rats in each group was 10.

The RES group’s brain TNF-α and PG-E2 levels were substantially greater than those of the controls (37.08 ± 0.84 ng/L and 637.50 ± 7.17 pg/ml, respectively; P-value less than 0.05 vs. 18.10 ± 0.85 ng/L and 424.33 ± 5.16 pg/ml). The RES + HNK group had a much lower P-value (less than 0.05) than the RES group (25.13 ± 0.80 ng/L, 524.50 ± 5.54 pg/ml). It was still significantly higher than the controls, though (P-value < 0.05) (Fig. 7A, B). Furthermore, compared to the control group, the RES group’s brain IL-10 levels were considerably lower (51.51 ± 1.44 vs. 94.41 ± 2.53 pg/ml, respectively; P-value less than 0.05). The values in the RES + HNK group were substantially higher than those in the RES group (75.65 ± 0.89 pg/ml; P-value < 0.05). The P-value was less than 0.05, which indicates that it was still significantly lower than the controls. The effect size was large regarding TNF- α, PG-E2 and IL-10 (0.991, 0996, and 0.992 respectively) (Fig. 7C).

### Gene expression analysis

**Fig. 8:**
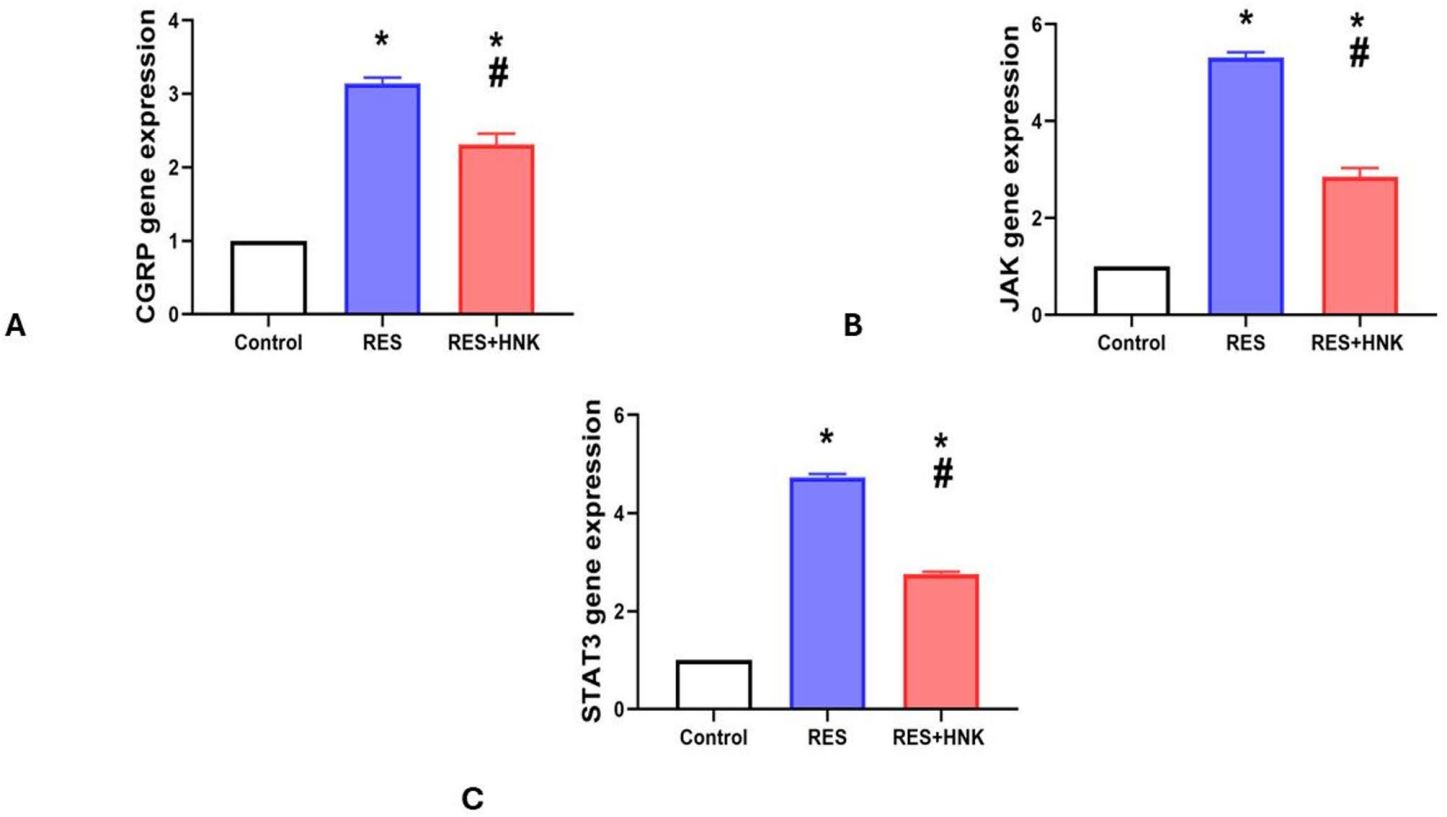
Honokiol impact on brain CGRP (**A**), JAK (**B**), and STAT3 (**C**) expression level in the RES group. Data are described as mean ± standard deviation (number equals ten). Group comparisons were made using the ANOVA test. * P-value is less than 0.05 vs. the control group. ^#^P-value is less than 0.05 vs. the RES group. *RES* reserpine, *HNK* honokiol. No of rats in each group was 10.

In comparison to the controls, the RES group’s brain CGRP, JAK, and STAT3 levels were greater (3.14 ± 0.08, 5.31 ± 0.11, and 4.72 ± 0.06 vs. 1.0 ± 0.00, respectively, P-value less than 0.05). The expression of the RES + HNK group was significantly lower than that of the RES group (2.31 ± 0.15, 2.58 ± 0.18, and 2.76 ± 0.04 respectively; P-value less than 0.05). It was still significantly higher than the controls, but (P-value less than 0.05). The effect size was large regarding brain CGRP, JAK, and STAT3 gene expression (0.990, 0.996, and 0.999 respectively) (Fig. 8).

### Correlation of CGRP with neurotransmitters, oxidative stress markers, and inflammatory markers

CGRP exhibited a strong negative correlation with dopamine, serotonin, and norepinephrine (*r* = − 0.988, − 0.987, and − 0.978; *P* < 0.05).

Additionally, CGRP demonstrated a strong positive correlation with MDA (*r* = 0.967; *P* < 0.05). Nonetheless, there was a strong negative correlation with SOD (*r* = − 0.985; *P* < 0.05).

**Fig. 9:**
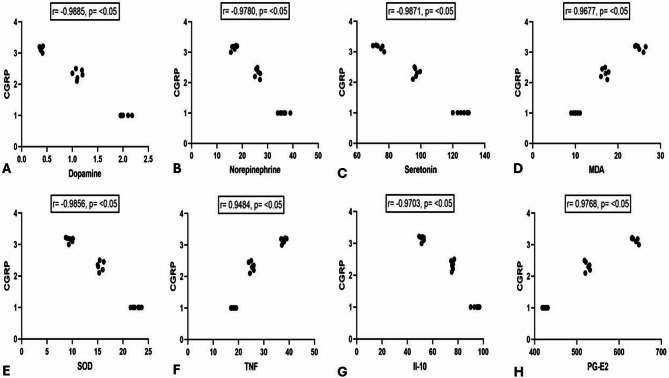
Pearson correlation between calcitonin gene receptor protein (CGRP) gene expression and Dopamine (ng/ml) (**A**), norepinephrine (nmol/L) (**B**), serotonin (ng/ml) (**C**), Malondialdehyde (MDA) (nmol/gr of tissue) (**D**), superoxide dismutase (SOD) (nmol/gr of tissue) (**E**), Tumor Necrosis Factor- α (TNF-α) (ng/L) (**F**), Interleukin (IL-10) (ng/L) (**G**), and prostaglandin E2 (PG-E2) (pg/ml)(**H**). No of rats in each group was 10.

Regarding inflammatory markers, CGRP displayed a strong positive correlation with TNF-α and PG-E2 (*r* = 0.948, and 0.976; *P* < 0.05). Conversely, CGRP exhibited a strong negative correlation with IL-10 (*r* = − 0.970; *P* < 0.05) (Fig. 9).

### Histopathological evaluation

#### In the H and E-stained

**Fig. 10:**
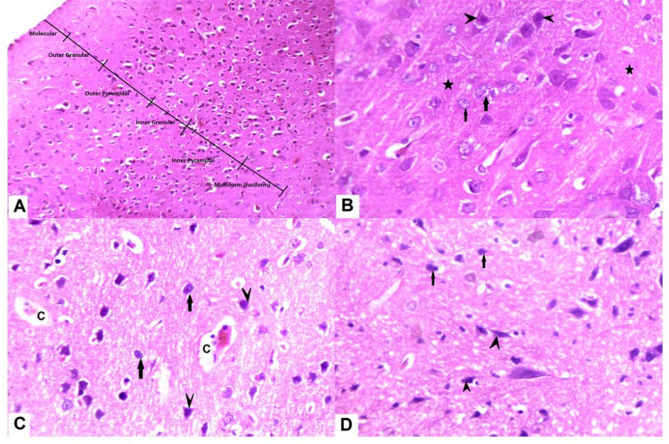
displays photomicrographs of the cerebral cortex stained with H&E. (**A**,**B**) The control group (**A**) exhibits six cerebral cortical layers. (**B**) The typical structure of the cerebral cortex shows acidophilic neuropil (star) that contains pyramidal (arrowhead) and granular (arrow) neurons. These neurons have large vesicular nuclei and basophilic cytoplasm. (**C**) The FM group has a significant presence of degenerated pyramidal neurons (arrow heads) and granular neurons (arrows) with condensed and darkly stained nuclei. Additionally, there is evidence of vacuolated neuropil (“v”) along with minor capillary congestion. (**D**) The RES + HNK group exhibits predominantly normal pyramidal and granular neurons, with a small number of degenerated granular neurons displaying pyknotic nuclei (arrows), as well as a few malformed pyramidal neurons (arrowhead). *RES* reserpine, *HNK* honokiol.

Slices of the cerebral cortex of control group, exposed nerve cell groups were organized into six well-defined layers: pia mater, outer granular layer, outer pyramidal layer, inner granular layer, inner pyramidal, and polymorphic layers. Pyramidal cells have open-face nuclei, lengthy apical dendrites, and basophilic cytoplasm. Spherical granular cells featured large vesicular nuclei with prominent nucleoli and homogenous eosinophilic neuropil between nerve cells. The RES group exhibited significantly higher apoptosis. Shrunken, irregular granular, and pyramidal cells were darkly stained with haloed and pyknotic nuclei. Neuronal and glial cell processes interact to generate homogeneous eosinophilic neuropil between nerve cells. The RES + HNK group demonstrated some intact pyramidal and granular neurons while exhibiting abundant apoptotic cells (Fig. 10).

#### Immunohistochemical analysis

**Fig. 11:**
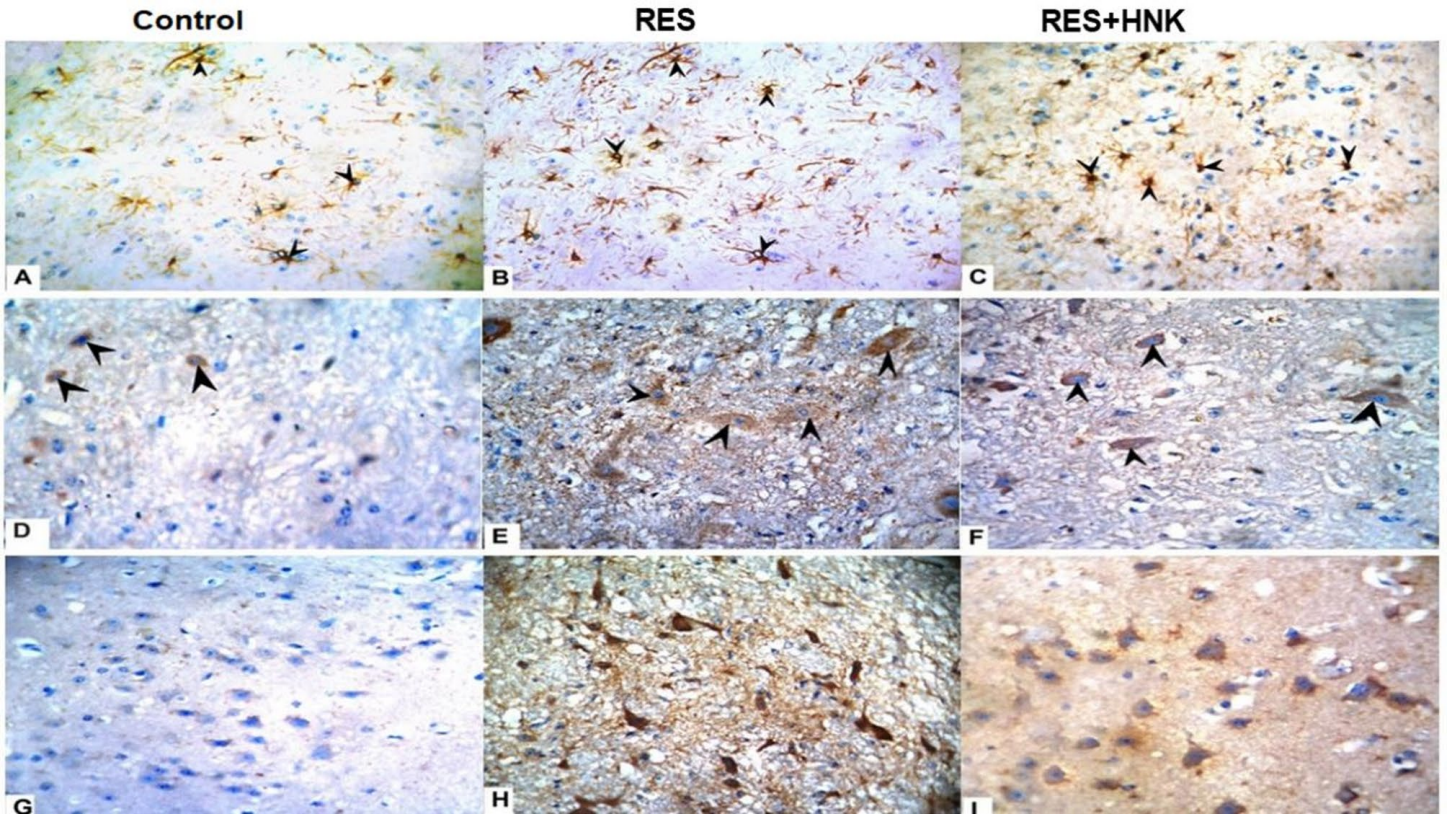
Honokiol’s effect on (**A**–**C**) GFAP response (astrocyte activation), (**D**–**F**) CD68 (microglia activation), and (**G**–**I**) Caspase 3 (apoptotic cells). An arrowhead is used to identify immunoreactive cells. The findings were plotted on a graph (**J**). Honokiol reduced the progression of fibromyalgia produced by reserpine by reducing GFAP expression in astrocytes, activated microglial cells, and caspase 3 (apoptotic cells) in the brain.

**Fig. 12:**
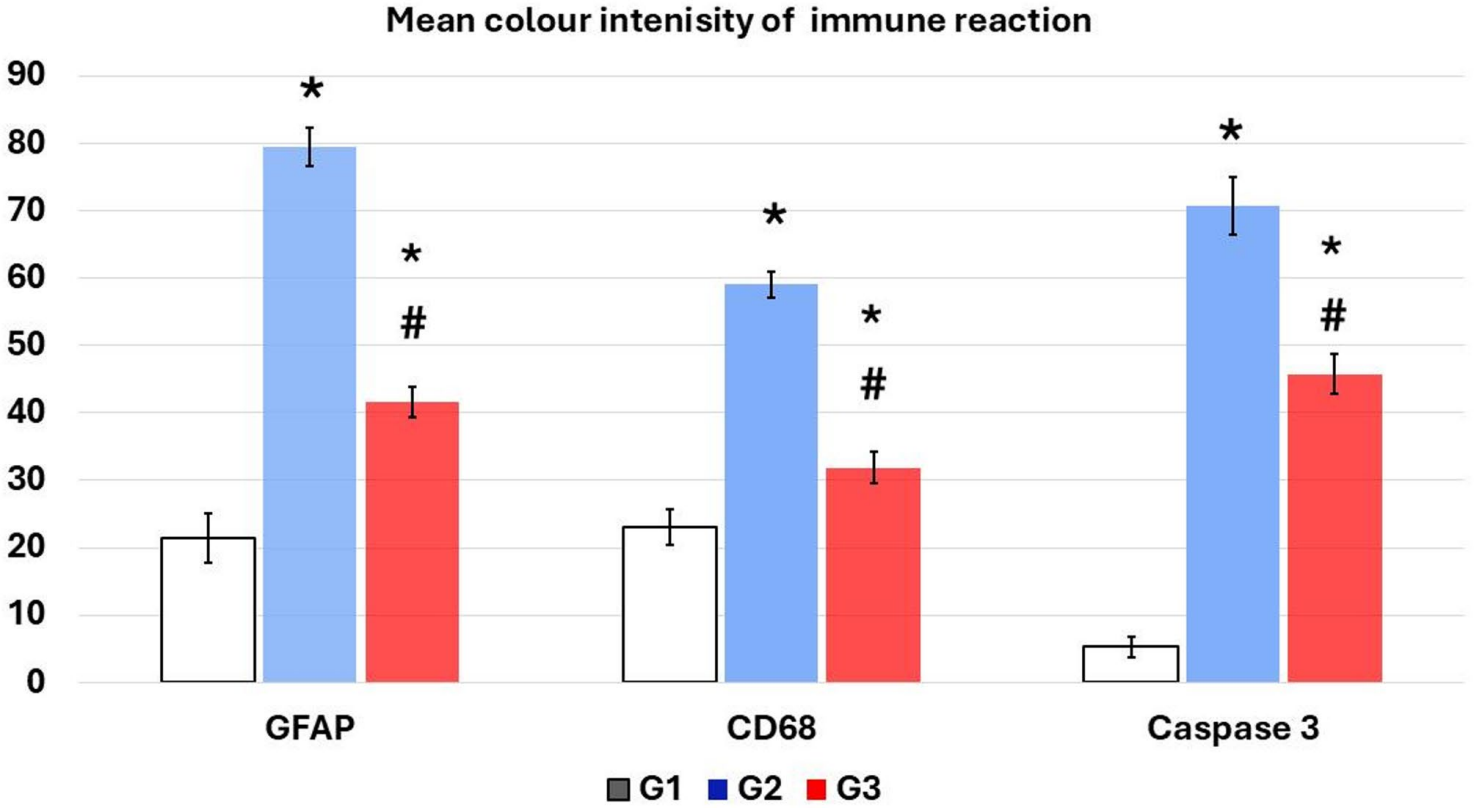
A chart demonstrating the mean color intensity of GFAP, CD68, and caspase-3 positive immunoreaction. *Data represented as mean ± SD (*n* = 10) and group comparisons were made utilizing the ANOVA test. G1: control group, G2: reserpine induced fibromyalgia group, and G3: honokiol treated group, *p < 0.05 is significant when compared to the control, and # *p* < 0.05 is significant when linked to the ROT group. No of rats in each group was 10.

An immunohistochemistry evaluation, conducted 21 days following the initial RES administration, revealed minimal reactivity and low levels of GFAP, CD68, and caspase-3 in the cerebral cortex of the control group. The RES rats showed significant immunoreactivity to GFAP, CD68, and caspase-3 in astrocytes, microglia, and nerve cells (*P* < 0.001, compared to control). HNK administration reduced GFAP, CD68, and caspase-3 levels compared to the RES group. However, they remained considerably greater than the control group (Figs. 11 and 12 and P-value less than 0.05). HNK administration significantly decreased the total number of glial cells, notably astrocytes and microglia, as well as apoptotic cells, compared to the reserpine-treated group (Table 1).

**Table 1:** Counts of astrocytes, microglia, and apoptotic cells.

	Astrocytes	Microglia	Apoptotic cells	*P* value
	Mean ± SD	Mean ± SD	Mean ± SD	
Control group	23.25 ± 1.77	3.95 ± 0.99	2.15 ± 0.875	
RES group	37.65 ± 1.53	7.65 ± 0.93	20.2 ± 1.96	P1 < 0.05*
RES + HNK group	30.50 ± 1.6	5.30 ± 0.92	13.05 ± 1.39	P2 < 0.05*
				P3 < 0.05*

*P value < 0.05.

P_1_ = comparison between RES group and control group.

P_2_ = comparison between RES + HNK group and control group.

P3 = comparison between RES group and RES + HNK group.

RES: reserpine, HNK: honokiol.

No of rats in each group was 10.

## Discussion

Chronic pain, affecting over 100 million Americans, surpasses the combined prevalence of cardiovascular disease, diabetes, and cancer. It’s characterized by an aberrant and non-protective response^31^. Defined as pain lasting over six months or beyond the typical tissue healing period, chronic pain is a significant health concern. FM is a chronic, widespread muscle pain condition. The prevalence of fibromyalgia in the United States and other countries is approximately 2–3%, with the incidence increasing with age^2^. FM is distinguished by morning stiffness, widespread pain sensitivity, and widespread discomfort, particularly in the trunk. FM is also linked to various additional symptoms, such as extreme exhaustion, psychological disorders (anxiety and/or depression), and sleep disruptions. The occurrence of comorbid symptoms changes with demographics, with pain and sleep difficulties affecting up to 90%, depression or anxiety in 40%, and fatigue impacting up to 100% of individuals with FM^32^.

While the exact etiology of FM remains elusive, it is evident that multiple systems are affected by FM. Proposed mechanisms underlying FM include central sensitization, muscle dysfunction, deficits in endogenous pain-modulating systems, and alterations in the hypothalamic-pituitary-adrenal axis^33^.

This study aimed to explore novel therapeutic approaches for effectively managing hyperalgesia, allodynia, and other FM manifestations.

In this research, the RES group exhibited a significant reduction in BW compared to the controls, likely due to decreased food intake associated with reserpine-induced depression, inflammation, and oxidative stress. This aligns with recent findings on body weight and muscle atrophy in reserpine-induced FM rats^34^. The study found that the RES + HNK group gained more weight than the RES rats, potentially attributed to lower levels of inflammatory markers including PG-E2, and TNF-α and elevated level of IL-10. Previous research by Chao et al. revealed that HNK reduced TNF-α release in mouse macrophages exposed to lipopolysaccharide, an inflammatory agent. Additionally, HNK inhibited NF-κB activation, protein kinase C-α membrane translocation, and nitric oxide expression in macrophages^35^.

Oxidative stress, characterized by an imbalance between the production of reactive oxygen species (ROS) and antioxidant defenses, has been implicated in the pathogenesis of various neurological disorders, including depression and anxiety^36^. FM patients have been shown to exhibit elevated levels of lipid peroxidation and reduced plasma antioxidant levels. Furthermore, mitochondrial dysfunction has been identified as a critical mechanism underlying chronic pain syndrome in FM^37^. The study revealed that RES rats exhibited significantly higher levels of MDA, a marker of lipid peroxidation, and lower levels of SOD, an antioxidant enzyme, compared to the control group rats. Reserpine, a known depleter of monoamines, can induce oxidative stress by autoxidation of dopamine and stimulation of oxidative catabolism via monoamine oxidase^37^. These processes can lead to the production of hydrogen peroxide and quinones in dopamine neurons, disrupting the redox status of their neuronal terminals, as evidenced by reduced levels of GSH and SOD^38^. Treatment with HNK, an active component of Magnolia officinalis extracts, resulted in increased SOD levels and decreased MDA levels in our study^39^. HNK is renowned for its potent antioxidant activity, being 1000 times more effective than α-tocopherol in inhibiting oxygen consumption and MDA formation through lipid peroxidation^11^.

In OFT, the RES group had significantly higher latency time and lower ambulation frequency than the controls. In the rotarod test, the time to fall decreased significantly in the RES group compared to the controls. These findings may be related to the dopamine depletion observed in this study. Yao et al. found reserpine-induced changes in the OFT and rotarod test in rats with marked reductions in fecal pellet output, ambulation, grooming, total locomotion, time spent on the rotarod, and rearing^40^. Muddapu et al. hypothesized that continuous reserpine treatment results in dopamine depletion in terminal endings of neurons through the regulation of magnesium- and ATP-dependent processes, ultimately causing monoamine depletion in the brain and a consequent decline in spontaneous locomotor activity, indicative of motor function deficits. In addition, depletion of biogenic amines (DA, 5-HT and NA), which results in increased nociception and depression in experimental rats^41^. HNK administration dramatically reduced latency time, enhanced ambulation frequency in OFT, and notably extended the time spent on the rotarod in the RES + HNK group compared to the RES group, which corresponded with a significant rise in dopamine levels. HNK has been found to have neuroprotective benefits, notably in the context of dopaminergic neuronal degeneration and motor deficits after unilateral striatal injection with 6-hydroxydopamine^11^. The neuroprotective effects of HNK may be attributed to the reduction of oxidative stress, activation of glial cells, and attenuate neuronal inflammation, as indicated by the decreased levels of inflammatory markers and caspase-3 expression in the cerebral cortex of HNK-treated group.

This study found that the immobility time in the FST was significantly greater in the RES group than in the controls, while the time spent of the EPM test was lesser in the RES group than in the controls, indicating anxiety and depression in this model. Yao et al. observed the same results, implying that depression is prevalent comorbidity in FM, alongside allodynia and hyperalgesia^40^. Nagakura and colleagues^16^ postulated that that repeated doses of reserpine lead to the depletion of biogenic amines in the nervous system, contributing to the development of sensory hypersensitivity as well as depressive- and anxiety-like behaviors. However, HNK administration significantly reduced immobility time in the FST, demonstrating its antidepressant effects through modulation of both the serotonergic and noradrenergic systems^42^.

In chronic nociceptive pain conditions, such as FM, the afferent fibers of the dorsal horn of the spinal cord are activated by noxious stimuli, resulting in the release of neurotransmitters and neuropeptides, which trigger a range of nociceptive and inflammatory responses that alter local neural pathways. This cascade of events results in heightened nociceptive and inflammatory responses, modifying local neural pathways and contributing to central sensitization. This phenomenon manifests as increased and persistent pain sensations, often accompanied by allodynia and hyperalgesia^43^. The RES model, a preclinical model of FM-like illness, recapitulates these core features, including allodynia, hyperalgesia, and behavioral changes^40^. In this experiment, RES administration induced nociception anomalies as evidenced by reduced treadmill endurance, delayed tail flick latency, and increased withdrawal scores in the paintbrush test. HNK therapy, by potentially reducing oxidative stress and inflammatory markers (TNF-α and PGE2) and elevation of IL-10 level, significantly ameliorated RES-induced hyperalgesia and allodynia. These findings align with previous research demonstrating the analgesic effects of HNK in pain models^39^. Furthermore, the modulation of neurotransmitters like dopamine, norepinephrine, and serotonin plays a crucial role in pain regulation within the CNS^44^.

Neurotransmitter analysis in this study revealed significantly lower levels of norepinephrine, serotonin, and dopamine in the RES group compared to controls. Reserpine, a known inhibitor of vesicular monoamine transporter 2, interferes with the storage and reuptake of monoamines in vesicles^40^. This depletion of neurotransmitters can lead to depression-like symptoms^41^. Dopamine has been implicated in pain modulation within the CNS, with dopamine receptor agonists demonstrating efficacy in reducing experimental hyperalgesia and allodynia^45^. Norepinephrine and serotonin play pivotal roles in the descending analgesia pathways from the midbrain periaqueductal gray matter to the spinal cord^46^. In FM patients, alterations in these neurotransmitters can contribute to noxious discomfort and exacerbate central pain symptoms^1^. The sustained reduction of monoamines supports the hypothesis that disruptions in monoaminergic regulation are implicated in the development of nociceptive pain, particularly in skeletal muscle and the CNS^16^.

RES group revealed significant elevation in JAK and STAT3 genes expression when compared to control group which may have a potential role in nociceptive mechanisms. Baral et al. concluded that the JAK/STAT pathway could also modulate anti-nociceptive mechanisms since IL-10, a potent anti-inflammatory cytokine, carries out its action through the JAK/STAT pathway^8^. In our study the RES + HNK group revealed significantly elevated IL-10 level as well as reduced expression of JAK and STAT3 genes when compared to the RES group.

In comparison with the control group, this research demonstrated that CGRP expression is upregulated in the RES group. Previous research has implicated CGRP, along with substance P, neurokinin-1 receptors, and NMDA-glutamate receptors, in the neurotransmission of pain^32^. Our study detected that CGRP exhibited a strong negative correlation with DA, serotonin, and NE. Hashikawa-Hobara et al. showed that injecting CGRP into the hippocampus significantly increases abundance MAOB and decreases hippocampal DA, thereby inducing anxiety-like behavior^47^. Our results detected that CGRP displayed a strong positive correlation with TNF-α and PG-E2. Conversely, CGRP exhibited a strong negative correlation with IL-10. Xiong et al. found that small interfering uc.48 + siRNA (Long noncoding RNA) could significantly decrease the high levels of IL-1β and TNF-α in the spinal cords and sera of rats with diabetic neuropathic pain. They guessed that uc.48 + siRNA treatment might down-regulate the expression of CGRP and then inhibit the release of proinflammatory IL-1β and TNF-α^48^.

The administration of HNK substantially reduced the expression of the CGRP gene in the RES + HNK group, suggesting a potential mechanism for its pain-relieving effects.

De Luca et al. found that anti-CGRP reduces oxidative stress biomarkers^49^. Furthermore, a study has demonstrated that CGRP can enhance the production of IL-6 in response to IL-1β or TNF-α, highlighting its role in inflammatory processes^6^.

To the best of our knowledge, this study is the first to investigate the effect of HNK on the expression of CGRP in RES-induced FM.

The RES + HNK group exhibited significantly elevated levels of dopamine, serotonin, and norepinephrine, potentially explaining the observed improvements in pain control (treadmill endurance, tail flick latency, and paintbrush tests) and behavioral measures of depression and anxiety (FST, and EPM).

This research found that FM increases glial cells, specifically microglia and astrocytes. FM activates the Janus-activated kinase/signal transducer activator of the transcription (JAK/STAT) pathway, leading to GFAP synthesis and astrogliosis^9,50^.

In chronic pain, microglia and astrocytes engage in a complex interplay, with nerve injury stimulating the production of CXCL13 and subsequent activation of astrocytes. This cascade of events results in the release of chemokines, pro-inflammatory cytokines, ATP, and glutamate, further activating microglia and contributing to neuronal hyperactivity. Additionally, pro-inflammatory cytokines produced by activated microglia can stimulate astrocytes, perpetuating the cycle of inflammation and pain^51^.

FM triggers apoptosis. The findings of this study corroborate the findings of Ribeiro et al., suggesting that nerve-ending TRP channels play a crucial role in neuronal injury-induced apoptosis. Calcium influx through TRP channels and oxidative stress, resulting from an imbalance between ROS production and antioxidant defenses, can contribute to this process^52^.

In this study, HNK therapy effectively reduced astrocyte and microglial activation, as well as caspase-3 activity. These findings are consistent with previous reports highlighting the neuroprotective effects of HNK, including its ability to suppress STAT3 activation and reduce GFAP expression^53^.

HNK exhibits neuroprotective properties by preventing oxidative damage, reducing brain excitotoxicity, attenuating neuroinflammation, and regulating mitochondrial function^11^. It achieves this by suppressing the expression of pro-inflammatory mediators, including TNF-α, IL-1β, iNOS, and IL-6, leading to a reduction in microglia and astrocyte activation^54^. The hydroxyl (-OH) groups present in HNK contribute to its potent antioxidant and free radical scavenging activities^11^. Furthermore, HNK has been shown to decrease ROS levels, improve mitochondrial membrane potential, enhance mitochondrial activity in brain cells, and elevate ATP levels^12^.

### Recommendations for future studies

Dose–response relationships: Include additional experimental groups with varying doses of honokiol to establish a clear dose-response relationship. Long-Term Follow-Up: Perform longer-term follow-up studies to assess the durability of honokiol’s therapeutic effects. Molecular Pathway Analysis: Include more detailed molecular pathway analyses to strengthen mechanistic conclusions. For example, investigate additional signaling pathways, such as NF-κB, MAPK, or TRPV1, which may contribute to FM pathophysiology. Positive Control Group: Add a positive control group using standard FM medications, such as pregabalin or duloxetine, to compare the efficacy of honokiol with existing therapies. Basal Nociceptive Sensitivity: Assess the basal nociceptive sensitivity of each rat prior to experiments to account for individual variability in pain sensitivity. Region-Specific Effects: Future studies should focus on region-specific effects in the brain and spinal cord, rather than using whole brain homogenates. This approach will better align with FM pathophysiology, as specific regions (e.g., the thalamus, amygdala, or periaqueductal gray) are known to play critical roles in pain processing and modulation. Combination Therapies: Explore the potential synergistic effects of honokiol in combination with existing FM medications or other natural compounds to enhance therapeutic outcomes. By addressing these recommendations, future studies can provide a more comprehensive understanding of honokiol’s mechanisms, efficacy, and therapeutic potential for fibromyalgia and related chronic pain conditions.

## Conclusion

This study presents robust evidence that honokiol (HNK) exerts significant anti-allodynic and anti-hyperalgesic effects in reserpine-induced chronic pain models, highlighting its potential as a therapeutic agent for fibromyalgia (FM). Notably, honokiol not only alleviated pain but also markedly reduced anxiety- and depression-like behaviors associated with FM. These beneficial effects were accompanied by a reduction in pro-inflammatory mediators, oxidative stress, pain-related neuropeptides, and apoptotic markers.

Mechanistically, honokiol demonstrated potent antioxidant and anti-inflammatory properties, evidenced by the elevation of interleukin-10 (IL-10) and the downregulation of JAK/STAT3 signaling pathways. Additionally, honokiol significantly reduced the expression of glial fibrillary acidic protein (GFAP) and CD68, indicating its ability to modulate glial activation and neuroinflammation. The downregulation of calcitonin gene-related peptide (CGRP) further underscores its role in mitigating pain sensitization.

Collectively, these findings suggest that honokiol alleviates FM symptoms through multifaceted mechanisms, including antioxidant, anti-inflammatory, and neuroprotective actions, positioning it as a promising therapeutic candidate for fibromyalgia and related chronic pain conditions.

## Data Availability

The datasets used and/or analyzed in the current investigation are accessible from the corresponding author upon reasonable request.
